# Menopausal symptoms by HIV status and association with health-related quality of life among women in Zimbabwe: a cross-sectional study

**DOI:** 10.1186/s12905-023-02466-1

**Published:** 2023-06-29

**Authors:** Tafadzwa Madanhire, Samuel Hawley, Ethel Dauya, Tsitsi Bandason, Ruramayi Rukuni, Rashida A Ferrand, Celia L Gregson

**Affiliations:** 1 The Health Research Unit Zimbabwe, Biomedical Research and Training Institute, 10, Seagrave Road, Harare, Zimbabwe; 2 SAMRC/Wits Developmental Pathways for Health Research Unit, School of Clinical Medicine, Faculty of Health Sciences, University of the Witwatersrand, Johannesburg, South Africa; 3 Infectious Disease Epidemiology, Faculty of Epidemiology and Population Health, London School of Hygiene and Tropical Medicine, London, UK; 4 Global Musculoskeletal Research Group, Musculoskeletal Research Unit, Bristol Medical School, University of Bristol, Bristol, UK; 5 Clinical Research Department, Faculty of Infectious and Tropical Diseases, London School of Hygiene and Tropical Medicine, London, UK

**Keywords:** HIV, Menopause, Menopausal symptoms, Quality of life, Africa, Ageing

## Abstract

**Background:**

The scale-up of antiretroviral therapy programmes has resulted in increased life expectancy of people with HIV in Africa. Little is known of the menopausal experiences of African women, including those living with HIV. We aimed to determine the prevalence and severity of self-reported menopause symptoms in women at different stages of menopause transition, by HIV status, and evaluate how symptoms are related to health-related quality of life (HRQoL). We further sought to understand factors associated with menopause symptoms.

**Methods:**

A cross-sectional study recruited women resident in Harare, Zimbabwe, sampled by age group (40–44/45–49/50–54/55–60 years) and HIV status. Women recruited from public-sector HIV clinics identified two similarly aged female friends (irrespective of HIV status) with phone access. Socio-demographic and medical details were recorded and women staged as pre-, peri- or post-menopause. The Menopausal Rating Scale II (MRS), which classified symptom severity, was compared between those with and without HIV. Linear and logistic regression determined factors associated with menopause symptoms, and associations between symptoms and HRQoL.

**Results:**

The 378 women recruited (193[51.1%] with HIV), had a mean (SD) age of 49.3 (5.7) years; 173 (45.8%), 51 (13.5%) and 154 (40.7%) were pre-, peri and post-menopausal respectively. Women with HIV reported more moderate (24.9% vs. 18.1%) and severe (9.7% vs. 2.6%) menopause symptoms than women without HIV. Peri-menopausal women with HIV reported higher MRS scores than those pre- and post-menopausal, whereas in HIV negative women menopausal stage was not associated with MRS score (interaction p-value = 0.014). With increasing severity of menopause symptoms, lower mean HRQoL scores were observed. HIV (OR 2.02[95% CI 1.28, 3.21]), mood disorders (8.80[2.77, 28.0]), ≥ 2 falls/year (4.29[1.18, 15.6]), early menarche (2.33[1.22, 4.48]), alcohol consumption (2.16[1.01, 4.62]), food insecurity (1.93[1.14, 3.26]) and unemployment (1.56[0.99, 2.46]), were all associated with moderate/severe menopause symptoms. No woman reported use of menopausal hormone therapy.

**Conclusions:**

Menopausal symptoms are common and negatively impact HRQoL. HIV infection is associated with more severe menopause symptoms, as are several modifiable factors, including unemployment, alcohol consumption, and food insecurity. Findings highlight an unmet health need in ageing women in Zimbabwean, especially among those living with HIV.

**Supplementary Information:**

The online version contains supplementary material available at 10.1186/s12905-023-02466-1.

## Background

People in Africa are now living longer than ever before; by 2025 the older population in Sub-Saharan Africa (SSA) is expected to have doubled from 2010 [1]. This ageing is attributed to improved living conditions, accessibility and availability of medicines such as antiretroviral therapy (ART), and means more women are now living through menopause [2].

Menopause is a physiological phase, defined retrospectively by 12 months of amenorrhea and loss of ovarian follicular function, typically occurring between the ages of 40 and 58 years [3]. The cessation of menstrual periods is often associated with a variety of adverse symptoms, including anxiety, mood disorders and vaginal dryness [4]. These symptoms begin as estrogen levels decline and may continue for years after menses end [4]. Although women experience similar hormonal changes, menopausal experiences vary greatly [5, 6]. Symptom severity is often evaluated using the validated menopause rating scale (MRS) II which characterizes psychological, somatic, and urogenital symptoms [7]. Severe menopausal symptoms can profoundly affect personal and social function [8], and health-related quality of life (HRQoL) [9–11], and are exacerbated by the effects of biological ageing, changing socio-demographics and comorbidities [12].

In Zimbabwe, 15.3% of adult women live with HIV and survival rates have greatly improved as a result of widespread ART roll-out [13]; hence increasing numbers of women with HIV are now reaching menopause. By contrast there is a paucity of data concerning the menopausal experiences of women living with HIV in SSA [14], with most data stemming from North American and European settings. However, one report from Nigeria suggests women with HIV may experience more menopausal symptoms than those without HIV [15]. Understanding menopause in a Zimbabwean population is important, as differences in culture, health-seeking behaviours, and availability of menopause care, invalidate extrapolation from high-income country settings [16, 17].

This study aimed to determine the prevalence and severity of self-reported menopause symptoms in Zimbabwean women at different stages of menopausal transition and determine how symptoms differ in the context of HIV infection. We further sought to explore other potential factors associated with menopausal symptoms, and to understand the extent to which menopausal symptoms relates to HRQoL in those with and without HIV.

## Methods

### Design and setting

A cross-sectional study of women in Harare, Zimbabwe was conducted between April and December 2020.

### Recruitment

Women, resident in Harare, were sampled by four age groups (40–44, 45–49, 50–54 and 55–60 years) and by HIV status. Women living with HIV were recruited from the Sally Mugabe (formally Harare Central) and Parirenyatwa Hospitals HIV clinics – the two public hospitals that serve the city of Harare. Women routinely attending the HIV clinic to collect their ART were invited to participate. Women living without HIV had been planned to be recruited from local churches in Harare. However, on the second day of recruitment, Zimbabwe implemented a lockdown in response to the COVID-19 pandemic and as a result the protocol was revised to adhere to infection prevention and control measures. Thus, women without HIV were recruited by asking each participant recruited from the HIV clinics to identify two female friends (irrespective of HIV status) around the same age with phone access who might be willing to take part.

### Inclusion criteria

The study included women aged 40–60 years, resident in Harare and who were willing to have an HIV test.

### Exclusion criteria

Women who were pregnant, acutely unwell or failed to provide informed consent were excluded from the study.

### Sample size calculation

Cochrane’s formula was used to calculate the power of the study (n = 378, p_1_ = 70.2%, p_2_ = 51.1%, z = 1.96, d = 0.05) [18]. The proportions of overall menopausal symptoms among women living with (p_1_) and without (p_2_) HIV were obtained from previous studies [19, 20]. The study had 84% power to detect a 15% difference in overall menopause symptoms between the two groups.

### Data collection

A female nurse-administered questionnaire recorded socio-demographic and lifestyle information, medical history, and menopause symptoms (see below). Socio-demographic factors included education, employment, household income and food insecurity (using selected questions from the US adult food security survey module [21]). Identified comorbidities included tuberculosis, diabetes, hypertension, cardiac disease, arthritis and balance impairment. Mental health was assessed using the validated 14-item Shona symptom questionnaire with women scoring ≥ 10/14 points classified as having mood disorders [22]. HRQoL was quantified using the WHOQOL eight-item questionnaire (score range: 1–40) which assessed perceived physical, mental and social health status over the previous two-weeks [23, 24]. The Global Physical Activity Questionnaire (GPAQ) quantified self-reported physical activity, from which minutes/week of moderate to vigorous intensity physical activity (MVPA) were calculated by summing moderate and vigorous work, travel-related and recreational activities (low physical activity defined as < 150 min/week of moderate to vigorous physical activity) [25].

Two nurses measured height (in cm) and weight (in kg) using a Seca 213 stadiometer and Seca 875 digital scales (Seca Precision for health, Seca Mechanical Floor Scales Class III, Hamburg, Germany) respectively, with the mean of both measurements calculated. Body mass index (BMI) was calculated (kg/m^2^) and categorized as underweight (< 18.5 kg/m^2^), normal (18.5–24.9 kg/m^2^), overweight (25-29.9 kg/m^2^) and obese (≥ 30 kg/m^2^) [26].

### Menopause status and symptoms

Participants were classified into one of three menopause categories based on self-reported final menstrual period (FMP) [27, 28]. Women currently having regular periods were classified as pre–menopausal. Women having irregular periods or having missed 3 months of consecutive menstrual periods within the past year, were classified as peri–menopausal. Women who had had no bleeding for more than 12 months were classified as post–menopausal. Women who had had a hysterectomy (+/- oophrectomy) could not be classified and were excluded from analyses. The menopausal rating scale II (MRS) was used to assess the severity of menopausal symptoms [no/little (0–4), mild [5–8], moderate [9–14, 29, 30] and severe (17+)], characterized by 11 questions measured on a five-point Likert scale. Three symptom sub-domains were rated: somatic (joint/muscle discomfort, sleep disturbance, heart discomfort, body temperature disturbance), psychological (irritability, anxiety, physical and mental exhaustion, mood disorders) and urogenital (vaginal dryness, urinary problems, sexual problems) [7]. The severity of MRS sub-domains were categorized into no/little, mild, moderate and severe groups according to sub-domain specific cut-offs, as previously described [7].

### HIV testing

All women recruited by participants with HIV had a point-of-care HIV antibody test performed using Alere Determine™ HIV-1/2 (Alere San Diego, Inc. San Diego, CA). If negative, there were enrolled into the study. If positive after a confirmatory test (Chembio SURE CHECK® HIV 1/2 Assay) they were enrolled into the HIV-positive group and referred to local HIV services [31].

### Statistical analysis

Data were cleaned, checked and analysed using Stata16 (StataCorp, College Station, TX). Unless otherwise stated, quantitative variables were summarised using the mean ± standard deviation (SD) if normally distributed, or otherwise as median with an interquartile range (IQR). Categorical variables were summarised as frequencies with percentages. Comparisons of quantitative variables by HIV status were performed using the Student’s t-test and the Mann Whitney U test if normally distributed or skewed respectively. Chi-squared and Fishers’ exact tests were used to compare categorical variables (including severity of overall MRS and the sub-domains) between women living with and without HIV. We evaluated whether the relationship between menopause stage and menopausal symptoms (and symptom sub-domains) was modified by HIV infection using a likelihood ratio test.

Univariate linear regression was used to determine the association between menopausal symptoms and HRQoL, reporting beta coefficients and their respective 95% confidence intervals. We tested whether the association between menopausal symptoms and HRQoL was modified by the presence of HIV infection using likelihood ratio testing. A test for trend was performed for HRQoL ranks across ordered total menopausal symptoms and menopausal symptoms sub-domains. Finally, univariate logistic regression was used to explore associations between socio-economic, obstetric, and maternal factors, as well as reported comorbidities, with menopausal symptoms (none/mild [MRS < 9], and moderate/severe [MRS ≥ 9]), reporting odds ratios (OR) with 95% confidence intervals.

## Results

### Participant characteristics

**Fig. 1:**
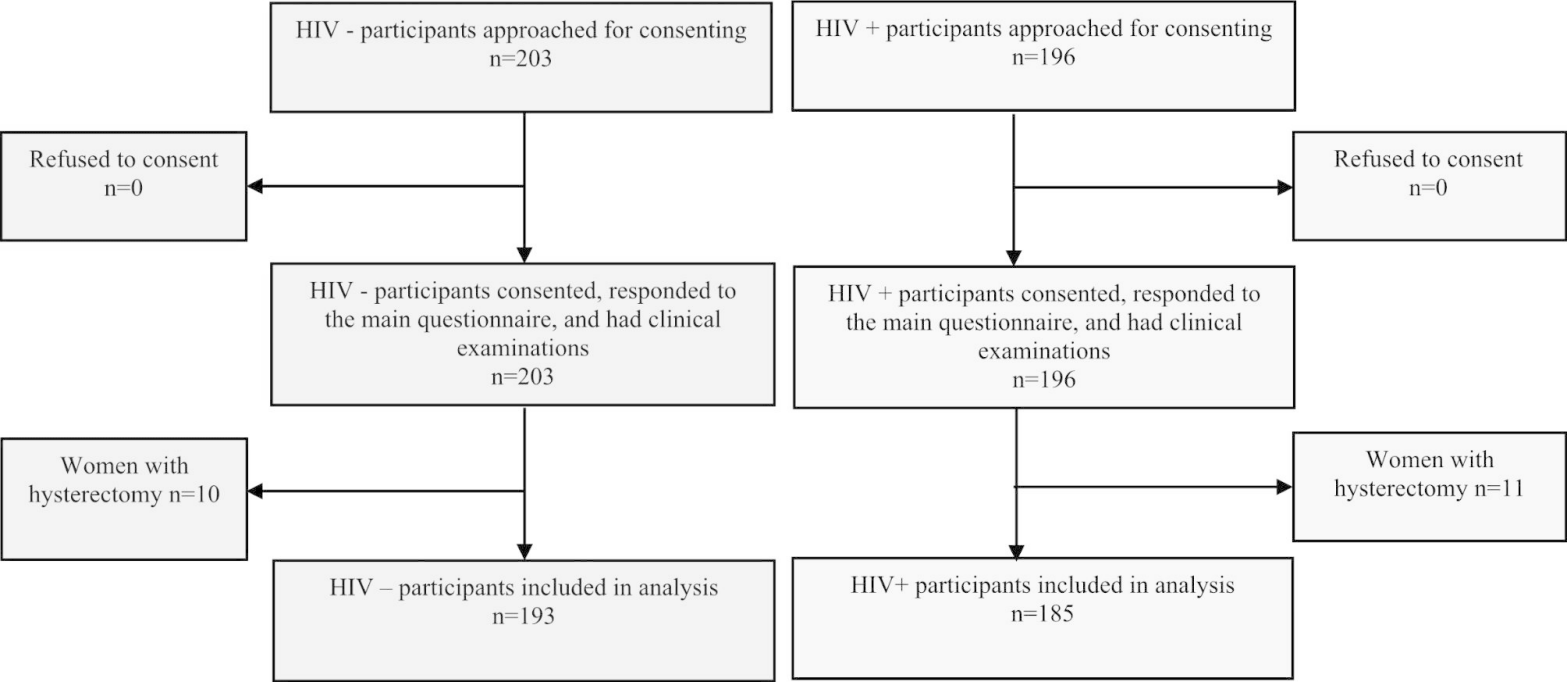
Participant recruitment flow diagram. Figure showing participant enrolment by HIV status

All 399 women who were approached consented to be recruited. After exclusion of 21 (5.3%) women who reported a hysterectomy (Fig. 1), analyses included 193 (51%) women with and 185 without HIV (Fig. 1); their overall mean (SD) age was 49.3 (5.7) years. Participant characteristics stratified by HIV status are reported in Table 1. Women living with HIV were frequently widowed (40.2%) or divorced/separated (25%), had lower educational attainment and were more often unemployed. Women living with HIV were less likely to report contraceptive use, reported bearing fewer children and were more often worried about food availability. Whilst the overall prevalence of obesity was high (37.8%), obesity was almost twice as common in women without HIV (26.5% vs. 48.7%). Correspondingly, whilst hypertension was common, identified in 31.6% of all women, it was less common in those living with HIV, than in those without HIV.

**Table 1:** Characteristics of women recruited in Harare, by HIV status

	Total (n = 378)	HIV – (n = 193)	HIV + (n = 185)	p value
**Demographic data**				
Age groups (years), n (%)				
40–44	95(25.1)	51(26.4)	44(23.8)	0.823
45–49	104(27.5)	51(26.4)	53(28.7)	
50–54	95(25.1)	46(23.8)	49(26.5)	
55–60	84(22.2)	45(23.3)	39(21.1)	
**Socio-economic characteristics**				
Highest level of education, n (%)				
None/Primary	57(15.1)	31(16.1)	26(14.1)	< 0.001
Secondary	258(68.3)	115(59.6)	143(77.3)	
Tertiary education	63(16.7)	47(24.4)	16(8.7)	
Employed, n (%)	194(51.3)	130(67.4)	64(34.6)	< 0.001
Household income ($US) per month, n (%)				
≤100	329(87.0)	168(87.1)	161(87.0)	0.352
101–200	34(9.0)	15(7.8)	19(10.3)	
>200	15(4.0)	10(5.2)	5(2.7)	
Worry of food insecurity in the household, n (%)	258(68.3)	120(62.2)	138(74.6)	0.010
Marital status, n (%) **				
Never married	6(1.6)	4(2.1)	2(1.1)	< 0.001
Currently married	181(48.0)	119(61.7)	62(33.7)	
Separated/Divorced	81(21.5)	35(18.1)	46(25.0)	
Widowed	109(28.9)	35(18.1)	74(40.2)	
Smoking history, n (%)				
Never	373(98.7)	192(99.5)	181(97.8)	0.340
Ex-smoker	4(1.0)	1(0.5)	3(1.6)	
Current	1(0.3)	0	1(0.5)	
Current alcohol uptake, n (%)	30(7.9)	14(7.3)	16(8.7)	0.616
**Obstetric and maternal factors**				
Age at menarche (years), n (%) **				
≤ 12	48(13.1)	18(9.5)	30(17.0)	0.164
13–15	204(55.7)	113(59.8)	91(51.4)	
≥16	114(31.2)	58(30.7)	56(31.6)	
Parity groups, n (%)				
Nulliparity	10(2.7)	2(1.0)	8(4.3)	0.046
Low parity (1–3)	229(60.6)	112(58.0)	117(63.2)	
Multi-parous (> 3)	139(36.8)	79(40.9)	60(32.4)	
Types of contraceptives, n (%)				
None	264(69.8)	125(64.8)	139(74.1)	0.033
Oral pill	72(19.0)	39(20.2)	33(17.8)	
Intrauterine device	15(4.0)	12(6.2)	3(1.6)	
Injectables (depo)	27(7.1)	17(8.8)	10(5.4)	
**Comorbidities**				
Number of comorbidities, n (%)				
None	201(53.2)	113(58.6)	88(47.6)	0.078
1–2	168(44.4)	77(39.9)	91(49.2)	
>2	9(2.4)	3(1.6)	6(3.2)	
Depression (Shona symptom questionnaire), n (%)	16(4.2)	5(2.6)	11(6.0)	0.105
Falls in the past year, n (%)				
None	336(88.9)	174(90.2)	162(87.6)	0.395
One	32(8.5)	16(8.3)	16(8.7)	
Two or more	10(2.7)	3(1.6)	7(3.8)	
Low moderate to vigorous physical activity (< 150 minutes/week)	19(5.0)	9(4.7)	10(5.4)	0.741
**HIV history**				
Years since HIV diagnosis, median (IQR)	10.0(5.8–14.0)	-	10.0(5.8–14.0)	-
ART duration (in years), median (IQR) **	9(5–13)		9(5–13)	-
**Anthropometry**				
BMI, n (%)				
Underweight	6(1.6)	0	6(3.2)	< 0.001
Normal	95(25.1)	35(18.1)	60(32.4)	
Overweight	134(35.5)	64(33.2)	70(37.8)	
Obese	143(37.8)	94(48.7)	49(26.5)	

Summary of participant’s socio-demographic characteristics, maternal, clinical history, and anthropometry measurements. The Chi-square test p-values were generated to compare categorical variables by HIV status. No comparisons were performed for HIV specific variables (ART duration and years after HIV diagnosis) ** represents complete case analysis for marital status (missing one participant), Age at menarche (missing 12 participants) and ART duration (missing eight participants)

Comorbidities were frequently observed; overall 52 (13.8%) women reported a history of tuberculosis, 119 (31.5%) hypertension, 18 (4.8%) diabetes, 16 (4.2%) cardiac disease, 5 (1.3%) poor balance, and 33 (8.7%) arthritis. Almost half of the women living with HIV had at least one comorbidity (Table 1). As expected, a history of tuberculosis was more common in women with HIV (50 [27.0%] vs. 2 [1.0%]; p < 0.001), whereas more women without HIV reported having a diagnosis of arthritis (26 [13.5%] vs. 7 [3.8%]; p = 0.001). No women reported to be breastfeeding.

### Menopausal symptoms

Most women were either pre- (n = 173, 45.8%) or post-menopausal (n = 154, 40.7%) (Table 2);. Similar proportions of women in each of the three menopausal stages were seen in those with and without HIV (Table 2). Overall, 140 (80.9%) pre-menopausal women had at least one menopausal symptom, as did 48 (94.1%) peri-menopausal and 119 (77.3%) post-menopausal women. Women with HIV were more likely to report moderate (24.9% vs. 18.1%) and severe (9.7% vs. 2.6%) menopausal symptoms, compared to women without HIV (Table 2). This pattern was also evident for the psychological and urogenital symptom sub-domains, with 25.4% of women with HIV reporting moderate or severe psychological symptoms, and 46.5% moderate or severe urogenital symptoms. However, there was no statistical evidence to suggest women living with HIV experienced more moderate or severe somatic symptoms (30.3% vs. 21.2%, p = 0.250, in those living with and without HIV respectively). In addition, HRQoL was no different between women living with and without HIV (Table 2).

**Table 2:** Menopausal stage, menopausal symptoms and health-related quality of life in women resident in Harare, by HIV status

	Total (n = 378)	HIV – (n = 193)	HIV + (n = 185)	p value *
**Menopausal stage**, n (%)				
Pre-menopause	173(45.8)	94(48.7)	79(42.7)	0.278
Peri-menopause	51(13.5)	28(14.5)	23(12.4)	
Post menopause	154(40.7)	71(36.8)	83(44.9)	
**Total MRS symptoms**, n (%)				
No, little (≤ 4)	177(46.8)	101(52.3)	76(41.1)	0.005
Mild (5–8)	97(25.7)	52(26.9)	45(24.3)	
Moderate (9–16)	81(21.4)	35(18.1)	46(24.9)	
Severe (≥ 17)	23(6.1)	5(2.6)	18(9.7)	
**Somatic symptom*sub-domain***, n (%)				
No, little (0–2)	190(50.3)	102(52.9)	88(47.6)	0.250
Mild (3–4)	91(24.1)	50(25.9)	41(22.2)	
Moderate (5–8)	82(21.7)	35(18.1)	47(25.4)	
Severe (≥ 9)	15(4.0)	6(3.1)	9(4.9)	
**Psychological symptom*sub-domain***, n (%)				
No, little (0–1)	228(60.3)	135(70.0)	93(50.3)	< 0.001
Mild (2–3)	75(19.8)	30(15.5)	45(24.3)	
Moderate (4–6)	53(14.0)	26(13.5)	27(14.6)	
Severe (≥ 7)	22(5.8)	2(1.0)	20(10.8)	
**Urogenital symptom*sub-domain***, n (%)				
No, little (0)	172(45.5)	100(51.8)	72(38.9)	0.009
Mild (1)	63(16.7)	36(18.7)	27(14.6)	
Moderate (2–3)	88(23.3)	35(18.1)	53(28.7)	
Severe (≥ 4)	55(14.5)	22(11.4)	33(17.8)	
**HRQoL**, mean (SD)	26.4(3.5)	26.5(3.3)	26.3(3.7)	0.642

Participants’ menopausal symptoms by HIV status. *p values generated using Chi-square tests of association for categorical variables and t-test for normally distributed continuous variables, MRS: Menopause Rating Scale, HRQoL: Health Related Quality of Life

Among women with HIV, those who were peri-menopausal reported higher MRS scores than those who were pre- and post-menopause, whereas in HIV negative women no clear association was seen between menopausal stage and MRS score (p-value for interaction = 0.014) (Table 3). Across the three menopausal symptom sub-domains, peri-menopausal women with HIV experienced the highest frequency of symptoms, with 78% experiencing somatic and/or urogenital symptoms, and 69% psychological symptoms. Pre-menopausal women with HIV also reported a high level of psychological symptoms (affecting 57%). Amongst women living with HIV, menopausal stage was strongly associated with the presence of somatic and psychological symptoms. A weaker relationship was seen between menopausal stage and somatic symptoms in women without HIV, with no relationship between menopausal stage and psychological or urogenital symptoms (Table 3). There was strong evidence for HIV infection modifying the association with menopausal stage and both somatic and psychological symptoms (interaction p-values 0.033 and 0.013 respectively), suggesting that women living with HIV experience more severe somatic and psychological symptoms through menopause.

**Table 3:** Menopausal symptom prevalence and severity across menopausal stages in women living with and without HIV in Harare

Menopausal symptoms	HIV negative (n = 193)				HIV positive (n = 185)				Interaction p-value
	Pre- (n = 94)	Peri- (n = 28)	Post- (n = 71)	p value	Pre- (n = 79)	Peri- (n = 23)	Post- (n = 83)	p value	
***Total***									
MRS II total, mean (SD)	4.4(4.6)	6.1(5.0)	5.6(4.9)	0.095	7.4(6.3)	11.4(7.8)	6.1(5.7)	0.004	0.014
Any symptoms, n (%)	70(74.7)	25(89.3)	53(74.6)	0.233	70(88.6)	23(95.8)	66(79.5)	0.029	
Any moderate or severe symptoms, n (%)	14(14.9)	7(25.0)	19(26.8)	0.147	27(34.2)	11(45.8)	26(31.3)	0.337	
***Somatic sub-domain***									
Sub-domain total, mean (SD)	2.1(2.2)	3.3(2.4)	3.2(2.9)	0.012	2.9(2.8)	4.8(2.8)	2.8(2.5)	0.010	0.033
Any symptoms, n (%)	36(38.3)	16(57.1)	39(54.9)	0.055	37(46.8)	18(78.3)	42(50.6)	0.027	
Any moderate or severe symptoms, n (%)	11(11.7)	8(28.6)	22(31.0)	0.005	22(27.9)	13(56.5)	21(25.3)	0.013	
***Psychological sub-domain***									
Sub-domain total, mean (SD)	1.3(2.0)	1.5(1.8)	1.1(1.6)	0.824	2.8(2.9)	3.8(3.6)	1.5(2.0)	0.001	0.013
Any symptoms, n (%)	28(29.8)	11(39.3)	19(26.7)	0.471	45(57.0)	16(69.6)	31(37.4)	0.006	
Any moderate or severe symptoms, n (%)	12(12.8)	6(21.4)	10(14.1)	0.516	25(31.7)	9(39.1)	13(15.7)	0.018	
***Urogenital sub-domain***									
Sub-domain total, mean (SD)	1.0(1.5)	1.4(1.9)	1.3(1.8)	0.494	1.6(1.9)	2.8(2.8)	1.8(2.4)	0.081	0.280
Any symptoms, n (%)	41(43.6)	16(57.1)	36(50.7)	0.393	48(60.8)	18(78.3)	47(56.6)	0.169	
Any moderate or severe symptoms, n (%)	25(26.6)	9(32.1)	23(32.4)	0.684	35(44.3)	16(69.6)	35(42.2)	0.058	

Comparison of menopausal symptoms by menopausal stage stratified by HIV status. MRS II: Menopause Rating Scale II; All continuous variables presented as mean (SD), Menopausal status (Pre-menopause, Peri-menopause, and Post-menopausal) All comparisons for categorical data presented were performed using the Chi-square test, Whilst means (SD) are presented for MRS II and sub-domain scores, Kruskal Wallis tests were used to compare medians across the menopausal stages given skewness of the data. Interaction test for the effect of menopause stage* HIV on menopausal symptoms

**Fig. 2:**
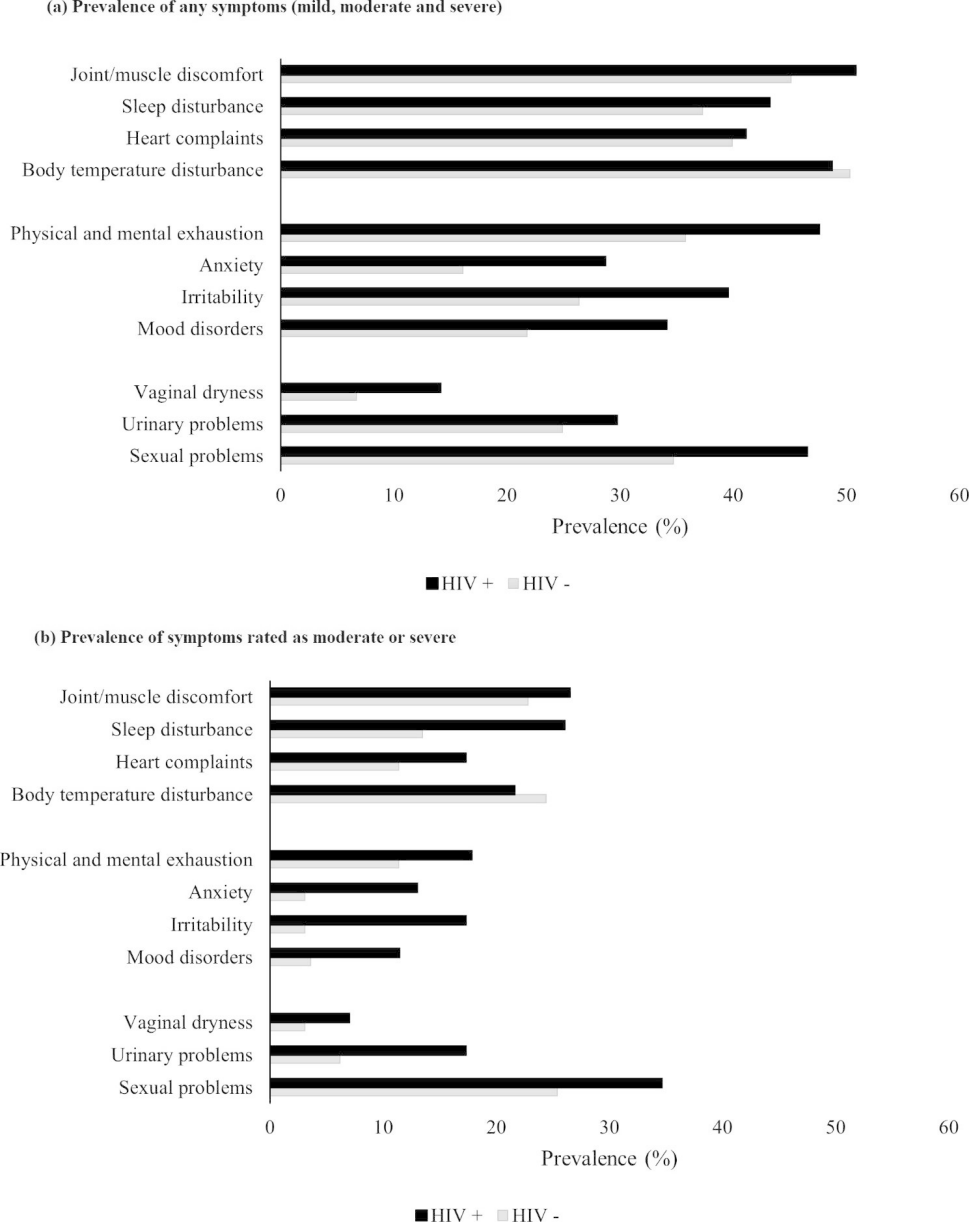
Prevalence of Menopause Rating Scale (MRS) II symptoms by HIV status in Zimbabwean women

Other than hot flushes, all menopausal symptoms, whether mild or moderate/severe, were more commonly seen in women living with HIV (Fig. 2). Generally, somatic symptoms were most commonly reported as affecting at least 40% of all women, as was physical and mental exhaustion (affecting ≥ 45%); however, moderate and/or severe sexual problems were a particular issue for women with HIV reported by 35%, as compared to 25% of women without HIV (Fig. 2b).

### Menopausal symptoms and HRQoL

Generally, with increasing severity of menopause symptoms (total MRS, somatic and psychological sub-domains), trends were seen towards lower mean HRQoL scores in both women with and without HIV (Table 4). Women with HIV who experienced moderate or severe symptoms reported substantially reduced HRQoL compared against those with few or no symptoms overall (based on total MRS score) and by all symptom sub-domains. The greatest impact on HRQoL was reported by those women with HIV experiencing severe somatic symptoms. In women living with HIV, clear associations were seen between moderate and/or severe psychological and urogenital symptoms and lower HRQoL, which were less evident in women without HIV. HIV infection did not appear to modify the association between menopausal symptoms (either total MRS or symptom sub-domains) and HRQoL (p > 0.1 for all) (Table 4).

**Table 4:** The association between menopausal symptoms and Health Related Quality of Life in Zimbabwean women living with and without HIV

Menopausal symptoms	HIV negative (n = 193)		p-value for trend	HIV positive (n = 185)		p-value for trend
	Mean (SD) HRQoL Score	Beta [95% CI]		Mean (SD) HRQoL Score	Beta [95% CI]	
**Total MRS score**						
No, little (0–4)	27.0(3.1)	Ref		27.1(3.5)	Ref	
Mild (5–8)	26.4(3.4)	-0.64 [-1.72, 0.45]	0.001	26.8(3.4)	-0.38 [-1.71, 0.96]	0.001
Moderate (9–16)	25.2(3.4)	-1.81 [-3.06, -0.56]		25.7(2.9)	-1.41 [-2.74, -0.09]	
Severe (≥ 17)	25.4(2.6)	-1.65 [-4.50, 1.21]		23.4(5.7)	-3.69 [-5.54, -1.83]	
**Somatic sub-domain**						
No, little (0–2)	27.3(2.8)	Ref		26.9(3.2)	Ref	
Mild (3–4)	26.2(3.6)	-1.09[-2.16, -0.02]	< 0.001	27.2(3.6)	0.36[-0.98, 1.69]	0.002
Moderate (5–8)	24.9(3.4)	-2.46[-3.67, -1.24]		25.2(3.9)	-1.67[-2.95, -0.40]	
Severe (≥ 9)	24.5(3.4)	-2.81[-5.42, -0.21]		22.6(4.7)	-4.33[-6.80, -1.86]	
**Psychological sub-domain**						
No, little (0–1)	26.8(3.1)	Ref		26.9(3.4)	Ref	
Mild (2–3)	25.4(3.5)	-1.41[-2.70, -0.12]	0.027	26.8(3.6)	-0.16[-1.46, 1.15]	0.004
Moderate (4–6)	26.1(3.5)	-0.77[-2.14, 0.61]		24.9(4.6)	-2.00[-3.56, -0.42]	
Severe (≥ 7)	24.5(3.5)	-2.34[-6.91, 2.22]		24.6(3.5)	-2.36[-4.14, -0.59]	
**Urogenital sub-domain**						
No, little (0)	26.7(3.6)	Ref		26.9(3.5)	Ref	
Mild (1)	26.4(3.1)	-0.24[-1.50, 1.02]	0.120	27.3(3.6)	0.34[-1.30, 1.98]	0.064
Moderate (2–3)	26.5(2.9)	-0.12[-1.39, 1.16]		25.7(3.1)	-1.18[-2.49, 0.13]	
Severe (≥ 4)	25.8(2.3)	-0.84[-2.37, 0.69]		25.2(4.8)	-1.67[-3.20, -0.15]	

*Beta [95% CI]: Univariate analysis of the association between menopausal symptoms on HRQoL. The univariate beta coefficients explain the difference in the effect of menopausal symptoms on HRQoL between different severity groups (mild, moderate and severe) when compared to the reference, with associated p-values. No evidence was detected for an interaction between menopausal symptoms and HIV infection on Health-Related Quality of Life for Total MRS or the symptom sub-domains*

### Associations with moderate/severe menopausal symptoms

Overall, 104 (18.8%) women reported moderate or severe menopausal symptoms. In addition to HIV described above, a number of other factors were associated with increased odds of moderate and/or severe symptoms (Table 5). By far the strongest association identified was for mood disorders (OR 8.80 [2.77, 27.3]), with other factors including history of two or more falls in the previous year (4.29 [1.18, 15.6]), early menarche (≤ vs. >12 years; 2.33 [1.22, 4.48]), alcohol consumption (2.16 [1.01, 4.62]), food insecurity (1.93 [1.14, 3.26]) and unemployment (1.56 [0.99, 2.46]). No association was seen between either BMI or comorbidities and moderate or severe menopausal symptoms (Table 5). In multivariate analysis, we identified consistent results though with attenuated associations between covariates and moderate/severe menopausal symptoms (Additional File Table 2).

**Table 5:** Factors associated with moderate and/or severe menopausal symptoms in Zimbabwean women living in Harare

	Association between potential risk factors and menopausal symptoms			p-value
	None / Mild (n = 274)	Moderate / Severe (n = 104)	Odds ratio [95% CI]	
**Socio-economic and household characteristics**				
Highest level of education				
None/Primary	40(14.6)	17(16.4)	Ref	
Secondary	184(67.2)	74(71.2)	0.94[0.50, 1.77]	0.863
Tertiary education	50(18.3)	13(12.5)	0.61[0.27, 1.41]	0.248
Employment status				
Employed	149(54.4)	45(43.3)	Ref	
Unemployed	125(45.6)	59(56.7)	1.56[0.99, 2.46]	0.054
Household income ($US) per month				
≤100	237(86.5)	92(88.5)	Ref	
1:101–200	24(8.8)	10(9.6)	1.07[0.49, 2.33]	0.858
2 > 200	13(4.7)	2(1.9)	0.40[0.09, 1.79]	0.229
Worry of food insecurity in the household	177(64.6)	81(77.9)	1.93[1.14, 3.26]	0.014
Marital status				
Currently married	130(47.6)	51(49.0)	Ref	
Separated/Divorced	62(22.7)	19(18.3)	0.76[0.41, 1.38]	0.361
Widowed	75(27.5)	34(32.7)	1.10[0.66, 1.83]	0.704
Never married	6(2.2)	0	-	-
Current alcohol uptake	17(6.2)	13(12.5)	2.16[1.01, 4.62]	0.047
**Obstetric and maternal factors**				
Age at menarche (years)				
≤ 12	27(10.1)	21(21.1)	Ref	
13–15	153(57.3)	51(51.5)	0.43[0.22, 0.82]	0.011
≥16	87(32.6)	27(27.3)	0.40[0.20, 0.82]	0.012
Parity				
Low parity (1–3)	169(61.7)	60(57.7)	Ref	
Nulliparity	7(2.6)	3(2.9)	1.21[0.30, 4.81]	0.790
Multi-parous (> 3)	98(35.8)	41(39.4)	1.18[0.74, 1.88]	0.492
Types of contraceptives, n (%)				
None	185(67.5)	79(76.0)	Ref	
Oral pill	56(20.4)	16(15.4)	0.67[0.36, 1.24]	0.200
Intrauterine device	15(5.5)	0	-	-
Injectables (depo)	18(6.6)	9(8.7)	1.17[0.50, 2.72]	0.714
**HIV history and self-reported comorbidities**				
HIV infection	121(44.2)	64(61.5)	2.02[1.28, 3.21]	0.003
Total number of comorbidities, n (%)				
None	153(55.8)	48(46.2)	Ref	
1–2	116(42.3)	52(50.0)	1.43[0.90, 2.26]	0.129
≥3	5(1.8)	4(3.9)	2.55[0.66, 9.88]	0.175
Depression (Shona symptom questionnaire)	4(1.5)	12(11.5)	8.80[2.77, 28.0]	< 0.001
Falls in the past year				
None	249(90.9)	87(83.7)	Ref	
Once	21(7.7)	11(10.6)	1.50[0.69, 3.24]	0.302
Twice or more	4(1.5)	6(5.8)	4.29[1.18, 15.6]	0.027
**Anthropometry**				
BMI				
Normal	68(24.8)	27(26.0)	Ref	
Underweight	3(1.1)	3(2.9)	2.63[0.50, 13.84]	0.254
Overweight	99(36.1)	35(33.7)	0.89[0.50, 1.61]	0.707
Obese	104(38.0)	39(37.5)	0.96[0.54, 1.69]	0.889
Low MVPA	16(5.8)	3(2.9)	0.48[0.14, 1.68]	0.250

*Determinants of moderate and severe menopausal symptoms in ageing women. The logistic regression model is presenting unadjusted odds ratios*

Nine women classified as post-menopausal reported using contraception (Additional File Table 1); 25 women reporting use of the oral contraceptive or the injectable depo reported moderate/severe menopausal symptoms, but no association between contraceptive use and menopausal symptoms was detected (Table 5). No woman reported use of menopausal hormone therapy (either vaginal or systemic oestrogens).

## Discussion

This study showed that menopausal symptoms are common in urban-dwelling Zimbabwean women as they transition through menopause, and that women with HIV experience more moderate and severe menopausal symptoms, (particularly somatic and psychological symptoms) than women without HIV. The most common menopausal symptoms in women living with HIV are joint/muscle discomfort, body temperature disturbance, physical and mental exhaustion and sexual problems. Somatic (joint/muscle discomfort, sleep disturbance, heart discomfort, body temperature disturbance), and psychological (irritability, anxiety, physical and mental exhaustion, mood disorders) symptoms were particularly associated with lower HRQoL.

Our study showed that menopausal symptoms are prevalent among Zimbabwean women before comparing to other studies. There are few studies of menopausal symptoms in African women; it has even been suggested that the warm climate may mask menopausal symptoms [5]. Religious and cultural norms among African women have historically been thought to provide a platform for positive adaptation to menopausal symptoms when they do occur [32]. However, a cross-sectional study of Nigerian women aged 40–60 years showed, as we have, that menopausal symptoms are common, particularly joint/muscle pain and hot flashes (somatic), physical and mental exhaustion (psychological) and sexual problems (urogenital) [33]. In Nigeria peri- and post-menopausal women experienced the more severe symptoms, although association with HIV status was not examined. In our study, 50% of Zimbabwean women reported body temperature disturbances. A small study of 118 rural-dwelling Xhosa women age 45–61 years in South Africa, reported hot flushes in 80% of women and night sweats in 92%; however, women were biased in selection having been sampled based on attendance at the medical practice of the author [34]. Our findings highlight the importance of making effective treatments available to women experiencing menopause. While oral and transdermal menopausal hormone therapy offers effective symptom management [35], availability is limited in SSA where economically challenged healthcare services tend to prioritise essential medicines [36].

Few studies have examined menopausal symptoms among women with HIV in Africa. We found that women living with HIV were particularly prone to somatic and psychological symptoms. A cross-sectional study of 714 Nigerian women, age 40–80 years, reported women living with HIV were three times more likely to experience severe menopause symptoms; however, the numbers with HIV were few [15]. Consistent with our study, cross-sectional studies from Brazil (251 women age 40 years and older) and Spain (251 women age 45–60 years) have reported an increase in somatic symptoms and vaginal dryness among women living with HIV, though the prevalence and severity varied according to socio-cultural and clinical characteristics [37, 38]. It has been hypothesized that somatic symptoms such as body temperature disturbances, may be worsened by ART or by HIV-associated immunological dysfunction affecting estradiol metabolism/ function and further research is needed [39].

We found that almost half of women living with HIV and a third of those without HIV reported sexual problems, 35% of women with HIV described these as moderate or severe, making this a common and debilitating symptom. Of the few other small studies of menopausal symptoms in African women none specifically addresses sexual problems [40, 41]. In our experience, sexual health of older women is rarely addressed as part of HIV care and therefore problems remain unidentified. We recommend that sexual health be routinely assessed in clinical practice and appropriate advice, counselling and treatment offered to women.

The study identified lower HRQoL in women with more frequent menopausal symptoms. The association we identified between overall, somatic and psychological menopausal symptoms and poorer HRQoL, is consistent with other studies from North and South America and Europe [42–44]. For example, the US National Health and Wellness Survey of women age 40–64 years, identified associations between mood disorders, anxiety and mood changes with reduced HRQoL to a similar extent as we have reported in Zimbabwean women [44].

Our study identified three potentially modifiable factors associated with menopausal symptom severity - alcohol consumption, falls and food insecurity. However, a causal relationship cannot be inferred from these cross-sectional data. Alcohol consumption and food insecurity were associated with twice the frequency of moderate to severe menopausal symptoms, while those who reported at least two falls in the past year were found to have a four-fold increase in symptoms. A dose-response association between alcohol consumption and menopausal symptom severity has previously been reported in American women [45]. Though alcohol consumption is frequently under-reported [46], plus a cross-sectional association may be explained by reverse causality since women with menopausal symptoms may consume alcohol in an effort to manage symptoms, e.g. of anxiety. Food insecurity is associated with sleep disturbance and mood disorders in older women [47, 48], both symptoms are included in the MRS score. Whilst these factors may not be causal, they are potential clinical indictors of women in whom more severe menopausal symptoms are reported.

Our study found older age at menarche to be associated with fewer moderate and severe menopausal symptoms. Though not previously reported in an African setting, a pooled analysis of studies from the UK, US and Australia identified an association between age at menarche and somatic menopausal symptoms, particularly body temperature disturbances, which may be explained by associations with BMI [49–51]. Whereas Iranian and Korean studies have reported conflicting results with an older age at menarche associated with menopausal symptoms [52, 53]. These inconsistencies may be explained by different racial, cultural and socio-economic backgrounds of women, variation in the outcome measure, and/or inconsistent use of menarche age thresholds.

### Strengths and limitations

Strengths of our study include a 100% participation rate despite a global pandemic, reflecting a desire to access health care for this otherwise neglected issue. In response to these findings, our research team conducted qualitative research to determine attitudes and understanding towards menopause, and went on to co-develop and co-design information resources on menopausal health with women in both South Africa and Zimbabwe, available in local languages [54]. Limitations include the cross-sectional study design which precludes conclusions on causal inference. We were unable to measure sex hormone levels to verify menopausal status. Although the MRS tool has been validated in Europe, America, and has been widely used in African settings, it has not been specifically validated in Zimbabwe, nor in people living with HIV. The study excluded women under 40 years of age, hence those with potential premature ovarian insufficiency were less likely to be studied.

## Conclusions

This study has shown that menopausal symptoms are common in older urban-dwelling Zimbabwean women as they transition through menopause, and women living with HIV tend to experience a greater prevalence of somatic and psychological symptoms. Menopausal symptoms understandably negatively affect quality of life. Our findings highlight an unmet health need in ageing women living with HIV in Zimbabwe. As populations age, HIV services are expected to need to manage growing numbers of women approaching menopause. Our findings suggest these services should routinely enquire of, and give advice on the management of, menopausal symptoms and the potential benefits of MRT use.

## Electronic supplementary material

Below is the link to the electronic supplementary material.

Additional File Table 1: Different contraceptive methods by menopause status in women living with and without HIV

Additional File Table 2: Multivariate model for factors associated with moderate and/or severe menopausal symptoms in Zimbabwean women living in Harare

## Data Availability

The datasets used and/or analysed during the current study are available from the corresponding author on reasonable request.

## List of abbreviations

SSA: Sub-Saharan Africa
HIV: Human Immunodeficiency Virus
ART: Antiretroviral Therapy
MRS: Menopause Rating Scale
HRQoL: Health-Related Quality of Life
WHOQOL: World Health Organisation Quality of Life Scale
GPAQ: Global Physical Activity Questionnaire
MVPA: Moderate to Vigorous intensity Physical Activity
BMI: Body Mass Index
SD: Standard deviation
IQR: Interquartile range
