# Supplementary material for: Menopausal symptoms by HIV status and association with health-related quality of life among women in Zimbabwe: a cross-sectional study
Source: BMC Womens Health. 2023 Jun 29;23:343. doi: 10.1186/s12905-023-02466-1 (PMC10311890; doi:10.1186/s12905-023-02466-1)
Supplement: Supplementary file 1 — Additional File Table 1: Different contraceptive methods by menopause status in women living with and without HIV [file 12905_2023_2466_MOESM1_ESM.docx]

**Supplemental Table 1: Different contraceptive methods by menopause status in women living with and without HIV**

|  | **Total**  **(n=378)** | **HIV –**  **(n=193)** | **HIV +**  **(n=185)** | **p value** |
| --- | --- | --- | --- | --- |
| Oral pill, n (%)  Pre  Peri  Post | 61(84.7)  8(11.1)  3(4.2) | 36(92.3)  3(7.7)  0 | 25(75.8)  5(15.2)  3(9.1) | 0.062 |
| Intrauterine device, n (%)  Pre  Peri  Post | 8(53.3)  4(26.7)  3(20.0) | 7(58.3)  3(25.0)  2(16.7) | 1(33.3)  1(33.3)  1(33.3) | 0.754 |
| Injectables, n (%)  Pre  Peri  Post | 18(66.7)  6(22.2)  3(11.1) | 10(58.8)  4(23.5)  3(17.7) | 8(80.0)  2(20.0)  0 | 0.506 |

*p-value: Comparison of different contraceptive methods use in women at different menopause stages by HIV status using a Chi-square test.*
