# Supplementary material for: Menopausal symptoms by HIV status and association with health-related quality of life among women in Zimbabwe: a cross-sectional study
Source: BMC Womens Health. 2023 Jun 29;23:343. doi: 10.1186/s12905-023-02466-1 (PMC10311890; doi:10.1186/s12905-023-02466-1)
Supplement: Supplementary file 2 — Additional File Table 2: Multivariate model for factors associated with moderate and/or severe menopausal symptoms in Zimbabwean women living in Harare [file 12905_2023_2466_MOESM2_ESM.docx]

**Supplemental Table 2: Multivariate model for factors associated with moderate and/or severe menopausal symptoms in Zimbabwean women living in Harare**

|  | | **Adjusted association between potential risk factors and menopausal symptoms** | | |
| --- | --- | --- | --- | --- |
|  |  | **Unadjusted odds ratio**  **[95% CI]** | **Adjusted odds ratio**  **[95% CI]** | **Adjusted**  **p-value** |
| **Socio-economic and household characteristics** | | | | |
| Highest level of education  None/Primary  Secondary  Tertiary education | | Ref  0.94 [0.50, 1.77]  0.61 [0.27, 1.41] | Ref  0.96 [0.44, 2.11]  0.93 [0.33, 2.69] | 0.918  0.900 |
| Employment status  Employed  Unemployed | | Ref  1.56 [0.99, 2.46] | Ref  1.15 [0.63, 2.10] | 0.649 |
| Worry of food insecurity in the household | | 1.93 [1.14, 3.26] | 1.79 [0.96, 3.31] | 0.065 |
| Current alcohol uptake | | 2.16 [1.01, 4.62] | 1.74 [0.69, 4.35] | 0.238 |
| **Obstetric and maternal factors** |  | | | |
| Age at menarche (years)  ≤ 12  13-15  ≥16 | | Ref  0.43 [0.22, 0.82]  0.40 [0.20, 0.82] | Ref  0.44 [0.21, 0.94]  0.35 [0.15, 0.81] | 0.034 |
| Parity  Low parity (1-3)  Nulliparity  Multi-parous (>3) | | Ref  1.21 [0.30, 4.81]  1.18 [0.74, 1.88] | Ref  1.87 [0.22, 15.9]  1.14 [0.65, 1.99] | 0.873 |
| **HIV history and self-reported comorbidities** |  | | | |
| HIV infection | | 2.02 [1.28, 3.21] | 1.71 [0.94, 3.11] | 0.076 |
| Total number of comorbidities, n (%)  None  1-2  ≥3 | | Ref  1.43 [0.90, 2.26]  2.55 [0.66, 9.88] | Ref  1.62 [0.94, 2.81]  5.05 [1.35, 18.9] | 0.009 |
| Depression (Shona symptom questionnaire) | | 8.80 [2.77, 28.0] | 7.05 [1.99, 24.9] | 0.002 |
| Falls in the past year  None  Once  Twice or more | | Ref  1.50 [0.69, 3.24]  4.29 [1.18, 15.6] | Ref  0.86 [0.33, 2.29]  5.01 [0.89, 28.0] | 0.205 |
| **Physical activity** |  | |  |  |
| Low MVPA | | 0.48 [0.14, 1.68] | 0.23 [0.04, 1.22] | 0.084 |

*The model is adjusted for highest level of education, employment status, household food insecurity, current alcohol intake, age at menarche, parity, HIV infection, comorbidities, depression, number of falls in the past year and moderate to vigorous physical activity (MVPA)*
